# ASO Author Reflections: Considerations in the Debate on Sentinel Node Biopsy in Older Patients with ER+/Her2− Breast Cancer

**DOI:** 10.1245/s10434-024-15095-1

**Published:** 2024-02-21

**Authors:** Alice Chung

**Affiliations:** Surgical Oncology, Cedars-Sinai Medical Center, Los Angeles, USA

## Past

Surgical management of the axilla in older women with favorable clinically node-negative breast cancer has been widely debated in recent years. In 2016, the Society of Surgical Oncology’s Choosing Wisely Recommendations were published advising against sentinel node biopsy (SNB) in women older than aged 70 years with hormone receptor-positive (ER+/Her2−) invasive breast cancer. These guidelines were based on randomized trials that demonstrated a lack of survival benefit in performing axillary dissection as well as retrospective studies that revealed no difference in survival with SNB omission.^1–4^ However, implementation of this practice has been met with challenges because of concerns regarding potential undertreatment and fears of recurrence. Additionally, there is a paucity of prospective data specifically addressing the utility of sentinel node biopsy in this patient population.

## Present

This single-institution, prospective study reports 3-year outcomes of 125 patients aged 65 years and older with clinical T1-2N0 ER+/Her2− invasive breast cancer treated with lumpectomy without SNB.^5^ Median age was 77 years, and median tumor size was 1 cm. Radiation therapy was performed in 29% of patients, and only 48% of patients were taking hormonal therapy at 2-year follow-up. Despite low utilization of adjuvant therapy, estimated 3-year outcomes were excellent with regional recurrence, disease-free survival, and overall survival rates of 98.2%, 91.2%, and 94.8%, respectively at median follow-up of 36.7 months. Noncompliance with hormonal therapy was the only factor on univariable analysis that was associated with any recurrence. This study provides prospective data that de-escalation of axillary surgery often is accompanied by de-escalation of adjuvant therapy and at 3 years is associated with low rates of recurrence or death. While this report was an interim analysis performed before reaching target accrual of 150, the findings thus far provide support for SNB omission in older patients with small ER+/Her2− tumors, although longer follow-up of more patients is critical to concluding the debate on this topic.

## Future

With accumulation of prospective data on this surgical approach, greater acceptance of the Choosing Wisely guidelines should be expected. However, the lack of compliance with adjuvant therapies remains a concern. As new treatment strategies for ER+/Her2− breast cancer, such as CDK4/6 inhibitors, emerge, compliance with adjuvant therapy may change, and indications for SNB may need to be reassessed. More than ever, the multidisciplinary collaboration addressing the preferences and tolerability of therapies in this patient population is paramount.
